# Spontaneous Haemorrhagic Gallbladder Rupture Causing Shock in a Background of Primary Sclerosing Cholangitis

**DOI:** 10.7759/cureus.82352

**Published:** 2025-04-16

**Authors:** Evangelia Florou, Shirin Ubaid, Matthew Seager, Michael Heneghan, Andreas Prachalias, Ameet G Patel

**Affiliations:** 1 Hepato-Pancreato-Biliary Surgery, King's College Hospital, London, GBR; 2 Hepato-Pancreato-Biliary Interventional Radiology, King's College Hospital, London, GBR; 3 Hepatology, King's College Hospital, London, GBR; 4 Hepato-Pancreato-Biliary Surgery and Liver Transplantation, London Bridge Hospital, London, GBR

**Keywords:** acute abdomen, gallbladder rupture, haemorrhagic gallbladder, haemorrhagic shock, primary sclerosing cholangitis

## Abstract

Haemorrhagic gallbladder (HG) is a very rare complication of acute cholecystitis. Cases are not frequently described in the literature, but frail elderly patients on anticoagulants are the primary group of patients affected. Semi-elective or emergency laparoscopic cholecystectomy is the treatment of choice, while haemorrhagic shock requiring emergency laparotomy is an extremely rare event. Primary sclerosing cholangitis (PSC) is an autoimmune liver disease (AiLD), and its association with bleeding complications remains a clinical observation that lacks robust scientific evidence and comprehensive understanding. Here, we present a case of gallbladder rupture causing haemorrhagic shock in a young patient with a background of cirrhotic PSC.

A 29-year-old female with PSC and a history of splenic artery aneurysm previously treated with embolization presented to the emergency department complaining of abdominal pain. A striking feature in the biochemistry results was the high bilirubin level without clinical detection of jaundice. Computed tomography (CT) showed a ruptured gallbladder with active bleeding and haemoperitoneum. Emergency laparotomy was mandated as the patient was becoming progressively haemodynamically unstable. The necrosed gallbladder was resected, and blood was evacuated from the abdominal cavity. The patient recovered well with no postoperative complications observed.

HG causing haemodynamic shock is quite a rare complication of acute cholecystitis. This is the first case reported in the literature of internal haemorrhage due to HG in a patient with a background of PSC. This case alerts clinicians dealing with PSC patients who may be susceptible to rare complications of common pathologies. AiLD and their relationship with bleeding events remains unclear and subject of future studies.

## Introduction

Haemorrhagic cholecystitis (HC) is a rare complication of acute cholecystitis with a reported incidence of 3.5% [1], ranging from 0.55% to 7% in the literature [2]. It is a rare cause of acute abdomen, causing haemorrhagic shock with reported high morbidity of 35-58% and mortality of 15-20% [1,3].

Identified risk factors are long-term usage of anticoagulants and antiplatelets [1,4,5], as well as non-steroidal anti-inflammatory agents [6]. Newly anticoagulated patients also seem to be at risk [7].

Presenting symptoms include right upper quadrant pain with positive Murphy’s sign, nausea, and vomiting. Gastrointestinal bleeding due to haemobilia has also been reported [8-10]. Catastrophic haemorrhage and haemodynamic instability may also be encountered, as in the case described here [9,11].

Primary sclerosing cholangitis (PSC) is an autoimmune liver disease (AiLD), a group of diseases that has been proven to be associated with a high incidence of antiphospholipid antibodies (aPL) positivity, thus increased tendency for thrombotic episodes [12]. On the other hand, the association of AiLD-related cirrhosis and bleeding events has been documented only in case reports [13,14]. PSC and its association with bleeding events is not scientifically proven; however, progression to cirrhosis may predispose to bleeding episodes [12,13].

Here, we present a case of spontaneous haemorrhagic gallbladder (HG) rupture causing haemorrhagic shock in a patient with a background of PSC.

## Case presentation

A 29-year-old female with a background diagnosis of crossover autoimmune hepatitis (AI) and PSC presented to the emergency department complaining about general malaise.

She had a known background of ulcerative pancolitis and backwash ileitis, for which she had undergone pan proctocolectomy, carrying a terminal ileostomy. She was also diagnosed with pyoderma gangrenosum, an extra-intestinal manifestation of her inflammatory bowel disease (IBD). Cirrhotic portal hypertension and oesophageal varices were under endoscopic surveillance. Furthermore, she had a history of spontaneous rupture of a splenic artery aneurysm previously treated with embolization, which resulted in functional asplenia. As PSC background is an additional factor to consider her immunocompromised, she was treated with lifelong antibiotic coverage with penicillin. The patient was under regular follow-up with the hepatology team for the last few years. Her baseline Child-Pugh score was A6.

The gallbladder had a thickened wall measuring 11 mm with no gallstones present. Histologically, most parts showed a preserved mucosa without significant inflammation, but there were areas of mucosal necrosis with haemorrhage. The wall was thickened with stromal oedema, haemorrhage, and neutrophilic infiltrate. The findings suggest localized mucosal necrosis leading to haemorrhage. Her clinical examination was unremarkable. Symptoms were those of fever and general malaise without abdominal pain. Her laboratory tests revealed raised inflammatory markers and mild derangement in liver biochemistry (Table 1). The working diagnosis was cholangitis, and she was treated with intravenous administration of antibiotics. She was discharged home after completing four days of admission with improvement noted in blood results and an additional seven-day course of oral antibiotics.

**Table 1:** Laboratory tests of the case on the first and second presentation. On first presentation, clinical and laboratory markers dictated cholangitis as the working diagnosis, in the absence of right upper abdominal pain, which would alert for gallbladder pathology. On the second presentation, the clinical absence of jaundice is in discordance with raised bilirubin, a fact that should have raised suspicions of haemobilia. WBC: white blood cell count; Hb: haemoglobin; CRP: C-reactive protein; Bil: bilirubin; AST: aspartate transaminase; ALP: alkaline phosphatase; GGT: gamma-glutamyl transferase.

	First presentation		Second presentation		Reference range
	Admission	Discharge	Admission	Discharge	
WBC	13.5	3.1	29	10	2.9-9.6 10*9
Hb	105	128	156 dropped to 96	118	115-135 g/L
CRP	54	17	67	30	<5 mg/L
Bil	83	38	158	30	1.7-20 umol/L
AST	41	39	77	59	10-35 IU/L
ALP	386	245	435	239	30-110 IU/L
GGT	240	143	387	148	5-40 IU/L

An ultrasound of the abdomen prior to discharge revealed mild thickening of the gallbladder wall with pericholecystic fluid indicative of early cholecystitis; however, the patient remained asymptomatic with no abdominal pain or positive Murphy’s sign during ultrasonography or on previous clinical examination by the bedside. There were no gallstones. As the patient was clinically and biochemically improving, she was found fit for discharge. In the absence of symptoms, ultrasonography findings of early acute cholecystitis were not assessed as significant enough to require a surgical opinion.

**Figure 1:**
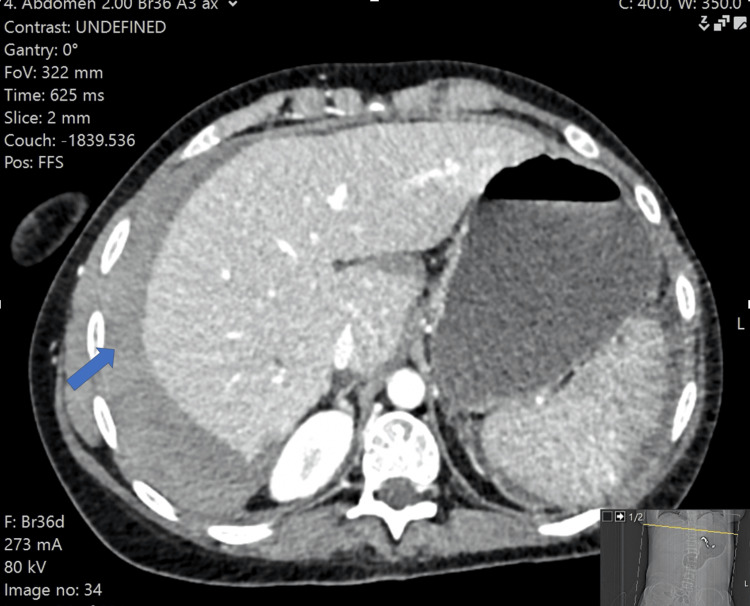
CT imaging of a case of haemorrhagic gallbladder. Haemoperitoneum with large perihepatic collection (arrow).

**Figure 2:**
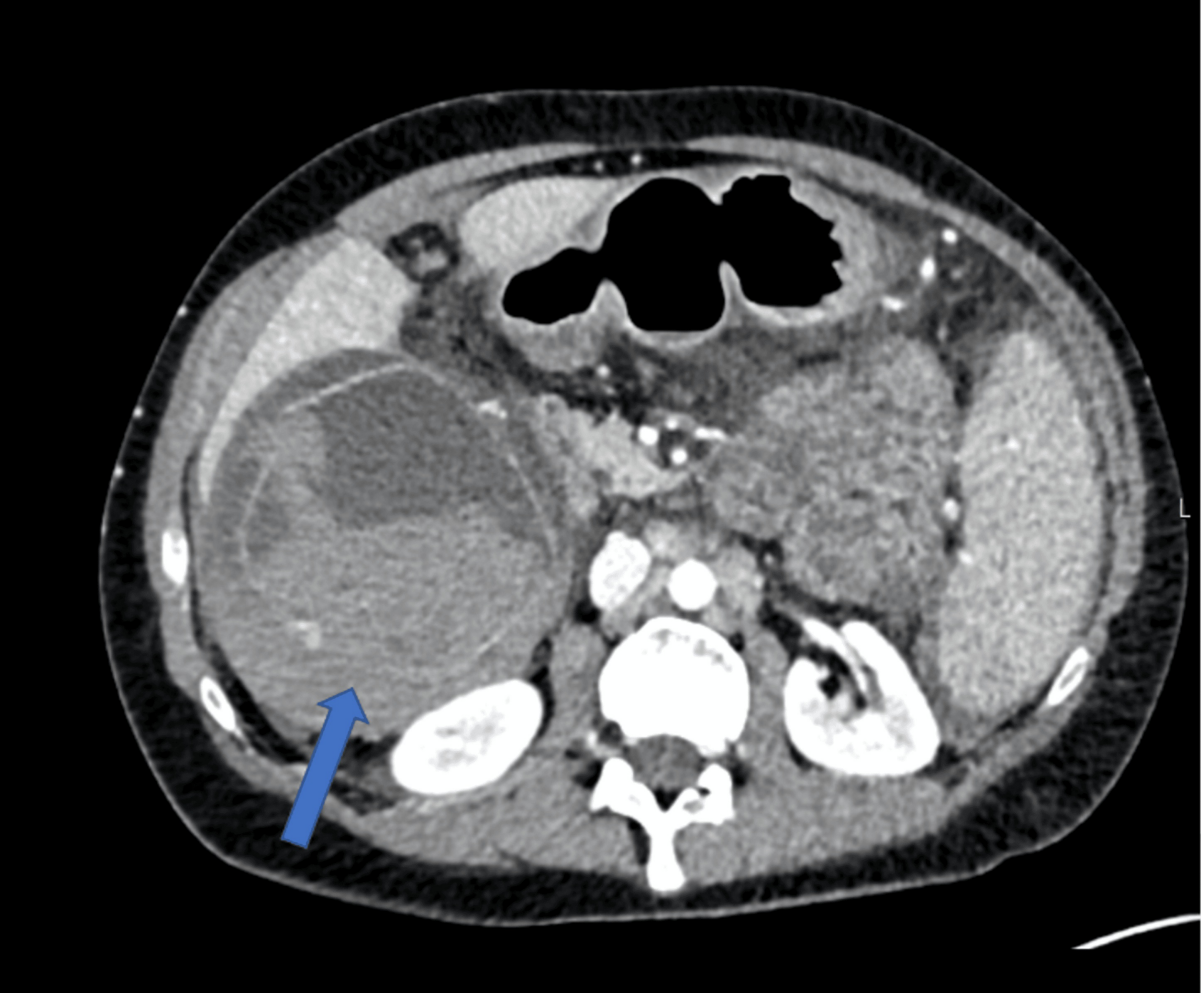
CT imaging of a case of haemorrhagic gallbladder. The gallbladder appears distended, containing a large amount of blood. A defect in the posterior gallbladder wall is noted, suggesting the site of rupture (arrow).

Four days after her discharge, she re-presented to the emergency department with the acute onset of abdominal pain in the right upper quadrant. The patient described the sharp and sudden onset of her pain, which felt familiar as it was similar to the one she experienced a few years ago at the time of her splenic aneurysm rupture. She was haemodynamically stable but mildly tachycardic and appeared clinically dehydrated. A striking feature in biochemistry was that of high bilirubin level without clinical detection of obstructive jaundice (Table 1). In the emergency presentation setting, this laboratory finding was not adequately taken into account or adequately explained. A first speculation of evolving liver decompensation with an acute thrombotic event involving the liver was made, and imaging was mandatory for further diagnosis. A computed tomography (CT) showed a ruptured gallbladder with active bleeding and an expanding haematoma surrounding the liver (Figures 1, 2).

After completing a three-hour stay in the emergency department and upon review of CT scan findings, aggressive resuscitation with intravenous fluid administration followed. On clinical re-review, the patient was becoming haemodynamically unstable, with shortness of breath requiring supplemental oxygen, complaining about worsening abdominal pain. On examination, she was found to have progressive abdominal distention. Repeat blood tests revealed a drop in haemoglobin (Hb) at 96 g/L, which required initiation of blood transfusion.

Emergency laparotomy was performed where a large amount of blood clots were evacuated. The necrotic gallbladder was resected after obtaining the critical view of safety, identifying both the cystic duct and artery. The patient recovered well with no postoperative complications observed. Ascites or any other signs of liver decompensation were not observed. She was discharged six days post procedure with improved liver biochemistry (Table 1).

**Figure 3:**
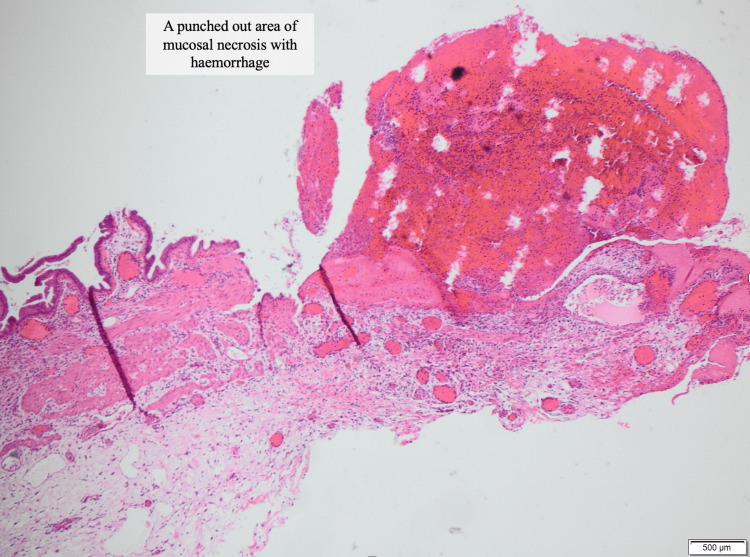
Histopathologic examination of haemorrhagic gallbladder. Microscopic appearance of haemorrhagic gallbladder (HG). The gallbladder mucosa with focal areas of necrosis.

Macroscopically, the gallbladder had a thickened wall measuring 11 mm with no gallstones. Histologically, most parts of the gallbladder wall showed a preserved mucosa without significant inflammation, but there were areas of mucosal necrosis with haemorrhage. The wall was thickened with stromal oedema, haemorrhage, and neutrophilic infiltrates. The findings suggested localized mucosal necrosis leading to haemorrhage (Figure 3).

## Discussion

HC is a rare complication of acute cholecystitis. The disease is scarcely reported in the literature and consists mainly of case reports and small case series [15]. The terminology is interchangeable between HC and haemorrhagic gallbladder (HG) [13,15].

The mechanism is poorly understood, but histopathological evidence shows transmural inflammation, which leads to ischemia and mucosal rupture. Gallstones may or may not be present [16].

In a literature review by Tarazi et al. in 2019, 45% of cases had the administration of anticoagulants as an identified risk factor for HC, and 45% reported as not being on anticoagulation, while in the rest 10% of the cases, there were no records available [15].

The disease appears to have the common initial symptoms of acute cholecystitis on presentation; however, it appears to have a spectrum as far as it concerns the evolution of symptoms, a factor that guides treatment. In the setting of sudden right upper quadrant pain and previous history of gallstones, episodes of cholecystitis or concurrent risk factors such as anticoagulation should raise suspicions for evolving HC.

Gastrointestinal bleeding due to haemobilia may also be encountered at a later stage [8,10], but hyperbilirubinemia may be the earliest finding of evolving haemobilia in the setting of HC, as was in this case. Abdominal pain, nausea, and vomiting may signify haemorrhage within the gallbladder, but it is unclear which cases could progress into haemodynamic instability and haemorrhagic shock. Hotak et al. reviewed gallbladder histology specimens of two centres over 21 years. The authors identified 35 cases of histopathologically characterised HG amongst 6458 cholecystectomies; thus, the incidence rate of HG was 0.55%. In that cohort, the gallbladder specimen was received from laparoscopic cholecystectomy, with only 11% of the cases reported as being converted to an open procedure. These results enhance the observation of the spectrum of the severity and evolution of this rare entity [2].

In the literature review by Tarazi et al., the authors reviewed 30 cases reported from 1985 to 2018. In this accumulative cohort, 16% of the cases were treated conservatively with intravenous antibiotics, 3% with naso-biliary drainage, and 10% with cholecystostomy [15]. Of the cases, 71% were treated with cholecystectomy. The urgency of the operation and the incidence of haemodynamic instability were not analysed.

The incidence of haemorrhagic shock caused by HG/HC is rarely reported [9,17]. Open cholecystectomy with or without preceding cystic artery embolization seems to be sufficient treatment in some cases [18]. Lan et al. reported a case of HG requiring emergency laparotomy in which left hepatectomy, cholecystectomy, and bile duct exploration were performed. In that case, initially the distended gallbladder mass wall appeared fistulating to the liver capsule, leading to spontaneous subcapsular rupture with haemorrhage, thus radiological appearances and course of disease were mimicking ruptured hepatocellular carcinoma, a working diagnosis which misled authors to proceed to extensive surgery [9]. The severity of the haemorrhage seems to define the management in cases with HG [9,15].

AiLDs have been strongly associated with a high incidence of positive antiphospholipid antibodies (aPL) in this group of patients, thus a tendency for thrombotic events [12,19].

Bleeding episodes have also been reported in the context of impaired coagulation when chronic liver disease and cirrhosis are established. Spontaneous retroperitoneal varix rupture, causing shock in a background of PSC cirrhosis [14], and spontaneous rupture of splenic aneurysm in the background of autoimmune liver cirrhosis have been documented, with the latter being included in the history of the case presented here [13]. HG in a background of autoimmune hepatitis and Child-Pugh B cirrhosis has also been documented [17].

On the contrary, documentation of bleeding events in the background of AiLD at early stages of the disease, when synthetic liver function is well preserved and clotting is within normal range, is limited in clinical observations with no scientific proof [13].

This is the first case reported in the literature of HG causing haemorrhagic shock in a patient with a previous history of a spontaneous bleeding event of splenic artery aneurysm rupture, in the background of PSC and early cirrhosis. The potential relationship of bleeding events and AiLD, as well as that of acute cholecystitis and spontaneous HG rupture leading to shock, remains undefined.

## Conclusions

HG is a rare, though well-documented, complication of acute cholecystitis. Clinicians should be aware of this rare entity as it can lead to acute abdomen and haemorrhagic shock, constituting a surgical emergency with high mortality rates reported in the literature. Hyperbilirubinemia can be a sign of haemobilia, and melena should be considered as a late symptom in the setting of evolving intrabiliary haemorrhage, causing gallbladder rupture and haemorrhagic shock.

Administration of anticoagulation and NSAIDs is a well-identified risk factor for HG. Progressive PSC can lead to cirrhosis, and the autoimmune nature of the disease, coupled with the development of cirrhosis, may create an environment leading to haemorrhagic complications. AiLD may be another risk factor for HG, and further studies are required to explain the relationship of this group of diseases with bleeding events.
